# The effect of *Bacillus coagulans* on cytotoxicity and apoptosis induced by *Salmonella* Typhimurium in HT-29 cell culture

**Published:** 2019-08

**Authors:** Amin Kawarizadeh, Farshad Nojoomi, Mohammad Tabatabaei, Saeid Hosseinzadeh, Mina Farzaneh

**Affiliations:** 1 Department of Microbiology, Faculty of Medicine, Aja University of Medical Sciences, Tehran, Iran; 2 Department of Pathobiology, Faculty of Veterinary Medicine, Shiraz University, Shiraz, Iran; 3 Department of Food Hygiene and Public Health, Faculty of Veterinary Medicine, Shiraz University, Shiraz, Iran

**Keywords:** Probiotics, *Bacillus coagulans*, Fluorescence staining, Minimum inhibitory concentration, *Salmonella* Typhimurium

## Abstract

**Background and Objectives:**

Human epithelial cells have been widely used to study the interaction between intestinal cells and pathogens, *in vitro*. In this study, the effect of probiotic bacteria *Bacillus coagulans* and its supernatant on the growth inhibition, cytotoxicity and induction of apoptosis caused by *Salmonella* Typhimurium and its adhesion to HT-29 cells were investigated.

**Materials and Methods:**

*B. coagulans* supernatant was used to obtain the minimum inhibitory concentration. To evaluate the cytotoxicity and percent of apoptotic cells, *B. coagulans* and its supernatant (2, 4, 6 and 8% concentrations) with *S.* Typhimurium was added to HT-29 cells. The MTT assay was used in order to evaluate the cytotoxicity. Percent of apoptotic cells was reported using a fluorescence staining method. Additionally, the adhesion of *S.* Typhimurium to HT-29 cells was investigated. The effect of *B. coagulans* on the level of adhesion was also studied.

**Results:**

The most inhibitory effect was shown at the concentration of 80000 μg/ml supernatant of *B. coagulans* (54.77% ± 1.43). The simultaneous culture of *S.* Typhimurium with *B. coagulans* had the lowest amount of cytotoxicity and induced apoptosis among the all co-culture groups of *S.* Typhimurium with *B. coagulans* or its supernatant. The determined cytotoxicity and induced apoptosis were 26.06 % ± 3.79 and 17.63 % ± 2.14 respectively. In the adhesion test, it was observed that *B. coagulans* can significantly prevent adhesion of *S.* Typhimurium to HT-29 cell.

**Conclusion:**

*B. coagulans* can reduce the adhesion, cytotoxicity and induction of apoptosis caused by *S.* Typhimurium in HT-29 cells *in vitro.*

## INTRODUCTION

According to the World Health Organization and FAO, probiotics are live microorganisms that have health benefits to the consumers, if consumed adequately (1). There is a strong tendency to use probiotic bacteria to treat and prevent various digestive disorders. Probiotics can inhibit the growth and invasion of pathogenic bacteria by strengthening the intestinal barrier and modulating the immune response by regulating the production of cytokines (2, 3). In addition probiotics can improve the intestinal microbial balance.

As such, microbial communities in the intestine or gut microbiome have a significant impact on the health of the host (4, 5). Human microbiome, especially intestinal microbiota, have a great impact on general health concerns and normal activities (6).

*B. coagulans* as a spore forming bacterium is one of the most promising probiotic bacteria, but still some of its pharmacological aspects have not been clarified yet (7). This bacterium can tolerate the acidic conditions of the stomach until it reaches the intestine and germinates there. It helps digest carbohydrates and proteins when it activates after germination in the gut (8). Consumption of *B. coagulans* makes the intestinal environment unfavorable for various pathogens and allows the use of nutrients by the intestinal system (9). In addition, studies have shown that *B. coagulans* I4, which is isolated from the cattle faeces, has antimicrobial activity with the production of bacteriocin-like inhibitors, called coagulin (10).

The usage of probiotics for the prevention and treatment of food poisoning has been considered. *Clostridium botulinum, Clostridium perfringens, Escherichia coli, Staphylococcus aureus* and *S*. Typhimurium are the major bacteria that cause foodborne illness (11).

In this research, *S*. Typhimurium has been studied. The genus *Salmonella* belongs to the *Enterobacteriaceae* family (12). This genus is composed of two species of *Salmonella enterica* and *Salmonella bongori*, a species of *Salmonella enterica*, containing more than 2000 different serovars. Some of these serovars, such as *Salmonella* Typhi, cause systemic infections and typhoid fever, and others, *S.* Typhimurium, cause gastroenteritis (13).

*S*. Typhimurium is an important food pathogen for both humans and animals (14). This organism has been associated with a wide spectrum of food contamination. *S*. Typhimurium, unlike *S.* Typhi, can also cause infection in many other mammalian species. Domestic animals can be used as a reservoir for the prevalence of this foodborne pathogen. This leads to a high incidence of non-typhoid *Salmonella* infection worldwide (13). *Salmonella* is capable of adhesion to host cells and invade and survive in the intestinal epithelial cells (15).

*In vitro* study on cell culture is an effective method for understanding the interaction between pathogenic bacteria and probiotics. This study was conducted to evaluate the antibacterial effects of *B. coagulans* on *S*. Typhimurium. For this purpose, the effect of *B. coagulans* and its supernatant on cell cytotoxicity and induced apoptosis by *S*. Typhimurium on the HT-29 cells was investigated using MTT and fluorescence staining. In addition, the minimum inhibitory concentration (MIC) of *B. coagulans* supernatant on this pathogen was also observed. The effect of *B. coagulans* and its supernatant on *S*. Typhimurium adhesion to HT-29 cells were evaluated.

## MATERIALS AND METHODS

### Bacterial preparation.

*Salmonella enterica* Serovar Typhimurium (*S*. Typhimurium) ATCC14028 was provided by the Department of Food Hygiene and Public Health, School of Veterinary Medicine, Shiraz University, Iran. *S*. Typhimurium was cultured in Tryptic Soy Broth (TSB) (Merck, Germany) and incubated for 24 hours at 37°C. The cell suspension was used to carry out strike culture on blood agar medium and was re-incubated similarly for overnight. Single colonies were used in subsequent tests.

### Cultivation of *B. coagulans* and preparation of its supernatant.

Frozen *B. coagulans* GBI-30 was provided by Department of Food Hygiene and Public Health, School of Veterinary Medicine, Shiraz University, Iran. The cells were cultured in TSB medium and incubated for 24 hours at 37°C. The cell suspension was then sub-cultured on the blood agar medium. Single colonies were used in subsequent tests. To obtain the supernatant from this bacterium, a colony was transferred to 10 ml of TSB and incubated for 24 hours at 37°C. The cell suspension was then transferred to 1000 ml of sterile TSB and re-incubated for 24 hours at 37°C. The cell suspension was centrifuged at 4000 rpm for 10 minutes. The supernatant was removed and dried by a freeze dryer machine. The supernatant deposition was prepared at concentrations of 1, 2, 4, 6 and 8% in RPMI 1640 medium and in 8% concentration in TSB medium. And finally filtered by 0.22 micrometer filter (16).

### Measurement of MIC.

Broth dilution method was used to measure MIC. For this purpose, 96-well microtitration plate (microdilution) was used. The 8% (80000μg/ml) supernatant in TSB medium in a volume of 100 μl/well and 4%, 2% and 1% concentration were prepared by serial dilution. Then, each well was inoculated with 10 μl of bacterial suspension (10^7^ CFU/ml). The TSB medium containing *S*. Typhimurium was considered as positive control and *B. coagulans* supernatant as negative control (17). Optical density of each well was measured at a wavelength of 600 nm at zero and after 24 hours after incubation at 37°C. The experiment was done in triplicate (18). The percentage of growth inhibition was calculated using the following equation (19):

$$\mathrm{growth}\;\mathrm{inhibition}=\frac{O-E}{O}\times 100$$

Where O means OD of positive control at hour 24 – OD at zero and E means OD of sample containing extract and bacterium at hour 24 – OD at zero

### HT-29 cell culture.

HT-29 cell line (Human colon adenocarcinoma) was provided by the Cancer Research Center, Shiraz University of Medical Sciences. HT-29 cells were cultured in 75 cm^2^ culture flask using RPMI 1640 medium with GlutaMax® (shell Max, Iran), 10% of heat-inactivated fetal bovine serum (Gibco Inc., America), 1% penicillin-streptomycin (10000 IU/ml and 10000 μg/ml) (Bio-idea Inc., Iran). The flasks were incubated in a humified incubator with 5% CO_2_ atmosphere at 37°C. Culture medium was replaced by fresh medium every 2 days (15).

### Preparation of cells for staining.

HT-29 cells were seeded into 12.5 cm^2^ flask at a density of 5×10^5^ cells /flask. The cells were incubated for 48 hours at 37°C under 5% CO_2_ atmosphere to reach the confluency of 90%. Then cultured *B. coagulans* and *S*. Typhimurium were centrifuged and the bacterial deposition was suspended in RPMI 1640 containing 2% fetal bovine serum. HT-29 cells were co-incubated with 2 ml of *B. coagulans* (10^7^ CFU/ml RPMI) plus *S*. Typhimurium (10^7^ CFU/ml RPMI), as well as 2 ml of each of 2, 4, 6 and 8% probiotic supernatant plus *S*. Typhimurium. The un-treated monolayer cells were considered as the negative control and the flask containing one layer of cells treated by *S*. Typhimurium was employed as the positive control. After 2 hours incubation, the supernatant was removed and then a cell washing with sterile PBS solution was done at 37°C. In order to harvest the cells from the 12.5 cm^2^ flask, 300 μl of filtered EDTA (1 mM) was used. The cells were collected via centrifugation and suspended in 1 ml of fresh RPMI 1640 medium.

### Evaluation of apoptosis by acridine orange and ethidium bromide staining.

Staining method was performed using the mixture of 1 μl of acridine orange (5 mg/ml), 1 μl of ethidium bromide (3 mg/ml) dissolved in 1ml of phosphate buffer solution (PBS). Then 20 μl of cell suspension was added to 5 μl of the dye solution. 10 μl of the dye-cell suspension was then placed on a hemocytometer and examined under a fluorescence microscope. Each sample was mixed just prior to microscopy and quantification. Acridine orange is a vital dye and stains both live and dead cells. Ethidium bromide stains only the cells that have lost membrane integrity. Live cells appeared uniformly green. Necrotic cells stained orange with no condensed chromatin. Apoptotic cells regarding be at the early or late stage of apoptosis, appeared green containing bright dots in nuclei or orange containing condensed nuclei, respectively. Experiment was performed in triplicates (20).

### Evaluation of cytotoxicity.

HT-29 cells were seeded in a 96-well flat bottom microtiter plate at a density of 10^4^ cells/well and incubated at 37°C in a CO_2_ incubator. The culture medium was replaced daily with 100 μl fresh RPMI 1640 medium containing 10% fetal bovine serum to reach a degree of confluency (about 90%.). 100 μl of each treatment were added to the wells containing a single layer of HT-29 cell line. After 2 hours of incubation, the culture medium was removed from wells. Subsequently, the MTT test was performed according to the manual of the manufacturer (Pars Tus Inc., Iran). Briefly, 100 μl of phenol red-free RPMI 1640 containing 2% fetal bovine serum and 10 μl of MTT working solution was added to each well and the plate was incubated for 4 hours at 37°C in a CO_2_ incubator. The medium was then aspirated, and the formazan crystals were solubilized by adding 50 μl of DMSO per well for 10 minutes at 37°C in a CO_2_ incubator. Finally, the intensity of the dissolved formazan crystals (purple color) was quantified using the ELISA plate reader at 570 nm. The test was done in triplicate. cytotoxicity was calculated by the following equation (16):

$$\mathrm{Cytotoxicity}=\left(1-\frac{\mathrm{OD}\;\mathrm{sample}}{\mathrm{OD}\;\mathrm{control}}\right)\times 100$$

### Bacterial adhesion assay.

HT-29 cells were cultured in the wells described in steps 4 and 5 in 24 wells cell culture plates (with 6 × 10^4^ seeding density). After formation of monolayer cell, 125 μl of each bacterial suspensions (2 × 10^7^ CFU/ml in RPMI medium) (at MOI of 10) and supernatant with different concentrations were added to wells and incubated for 2 hours. Then culture medium was removed and the HT-29 cells washed three times using PBS (15). Then the epithelial cells were lysed with 0.1% (v/v) Triton X-100 (Merck, Germany) for 5 min at 37°C (21, 22). In order to record the adhered viable bacteria, the suspension was cultured in the nutrient agar medium. After one day incubation, the formed colonies were transferred to MacConkey agar media by replica plating technique. To differentiate between *B. coagulans* and *S*. Typhimurium, colonies that were grown on the nutrient agar and MacConkey agar were considered as *S*. Typhimurium and colonies that were grown on nutrient agar but did not grow on the MacConkey agar were considered as *B. coagulans*. Each experiment was performed in triplicates.

### Statistical analysis.

Statistical analysis was performed using the SPSS software version 16 (SPSS, Inc., Chicago, IL), one-way ANOVA and Tukey post hoc tests were used. All results were shown as mean standard deviation. Differences at p<0.05 were considered as statistically significant.

## RESULTS

### MIC.

**Fig. 1:**
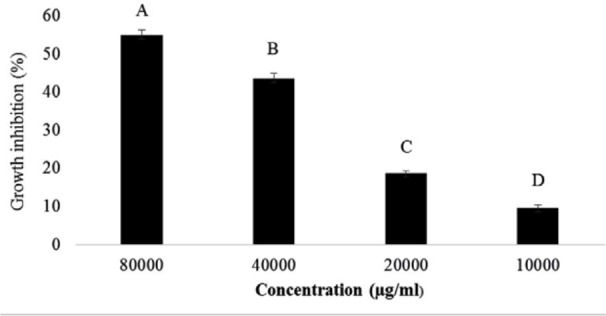
Minimal inhibitory concentration of *B. coagulans* supernatant against *S*. Typhimurium. The antennas represent the standard deviation value and different Latin capital letters above the columns indicate a statistically significant difference between the columns (p<0.05)

Results showed, 8% and 4% concentrations of supernatant (80000 and 40000 μg/ml) had the highest inhibitory effect on the growth of *S*. Typhimurium (with the values of 54.77% ± 1.43 and 43.46% ± 1.32, respectively) (Fig. 1).

### Cell cytotoxicity.

**Fig. 2:**
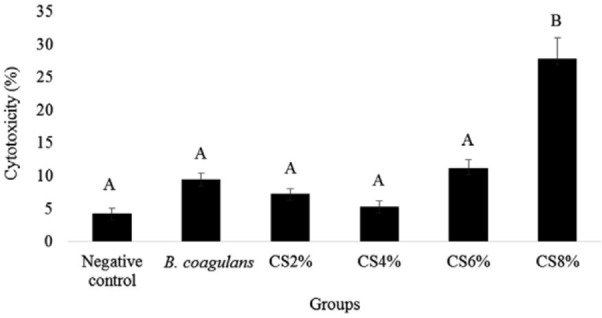
Cytotoxicity of *B. coagulans* and its supernatant with various concentration (CS). The antennas represent the standard deviation value and different Latin capital letters above the columns indicate a statistically significant difference between the columns (p<0.05)

MTT assay was used to study the effects of *B. coagulan*s on the cytotoxicity of *S*. Typhimurium. The highest and lowest cytotoxicity was observed in the 8% and 4% concentrations with the averages of 27.86±3.09 and 5.31±0.83, respectively. However, only the 8% concentration showed a significant level of cytotoxicity (Fig. 2).

**Fig. 3:**
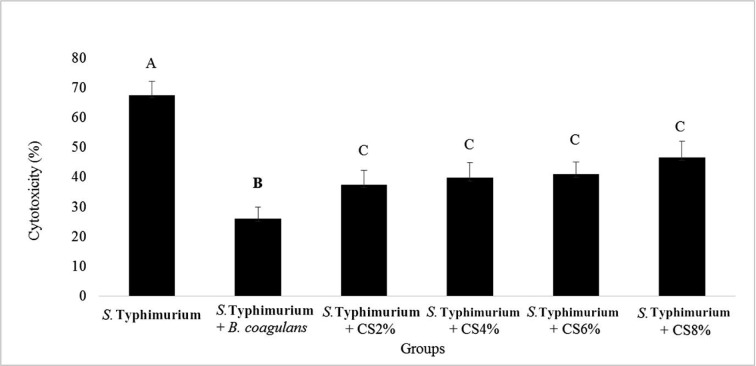
Cytotoxicity of *S*. Typhimurium alone and with *B. coagulans* and its supernatant (CS) with various concentration. The antennas represent the standard deviation value and different Latin capital letters above the columns indicate a statistically significant difference between the columns (p<0.05)

Concomitant culture of HT-29 cells with *S*. Typhimurium alone, together with *B. coagulans* or its supernatant were also performed. The highest and lowest cytotoxicity were respectively obtained for *S*. Typhimurium alone and its simultaneous culture with *B. coagulans* (with the averages of 67.61±4.51 and 26.06 ± 3.79). In these experiments, simultaneous culture of *S*. Typhimurium and *B. coagulans* was remarkably reduced the cell cytotoxicity caused by *S*. Typhimurium (Fig. 3).

### Induction of apoptosis.

**Fig. 4:**
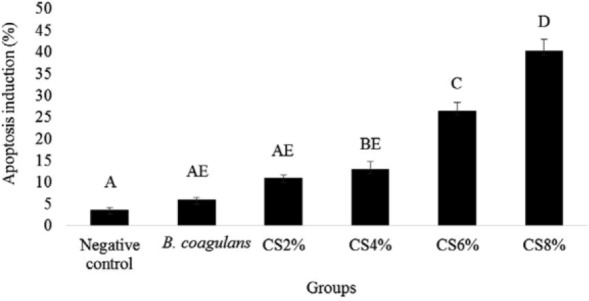
Apoptosis induction caused by *B. coagulans* and its supernatant (CS). The antennas represent the standard deviation value and different Latin capital letters above the columns indicate a statistically significant difference between the columns (p<0.05)

Fluorescence staining using acridine orange and ethidium bromide was used to study the effects of *B. coagulans* on the induction of apoptosis in the HT-29 cells caused by *S*. Typhimurium. The results showed that *B. coagulans* and its supernatant revealed the highest and lowest amount of apoptosis induced in HT-29 cells was recorded for *B. coagulans* and its 8% supernatant with the averages of 6.01 ± 0.42 and 40.25 ± 2.66 (Fig. 4).

**Fig. 5:**
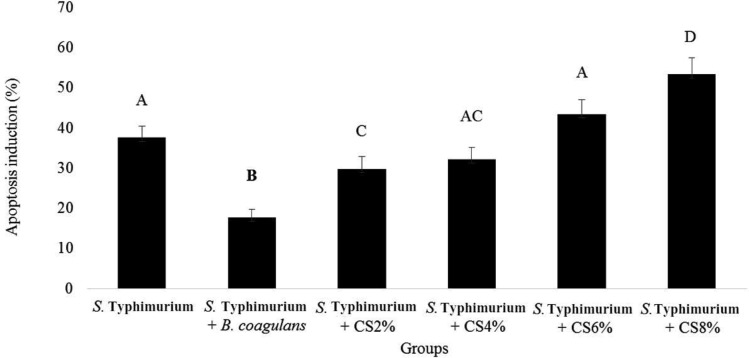
Apoptosis induction caused by *S.* Typhimurium alone and with *B. coagulans* or its supernatant (CS). The antennas represent the standard deviation value and different Latin capital letters above the columns indicate a statistically significant difference between the columns (p<0.05)

The simultaneous cultures of HT-29 cells with *S*. Typhimurium, *B. coagulans* or its supernatant revealed the highest and lowest percent of induction of apoptosis in *S*. Typhimurium with 8% supernatant and simultaneous culture of *S*. Typhimurium with *B. coagulans* (with the averages of 53.26 ± 4.13 and 17.63 ± 2.14) (Fig. 5). The results showed that *B. coagulans* significantly reduced the induction of apoptosis in HT-29 cultured cells induced by *S*. Typhimurium (Fig. 5).

### Bacterial adhesion assay.

**Fig. 6:**
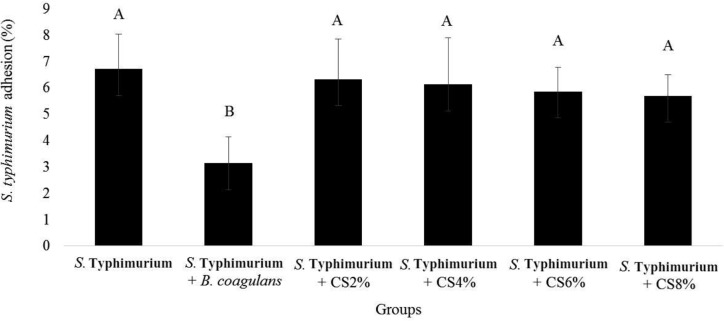
Percent of *S*. Typhimurium adhesion to HT-29 cells, alone and with *B. coagulans* and its supernatant (CS). The antennas represent the standard deviation value and different Latin capital letters above the columns indicate a statistically significant difference between the columns (p<0.05)

The effect of *B. coagulans* and its supernatant on adhesion of *S*. Typhimurium to the HT-29 was investigated. Results showed the highest level of adhesion (6.71 ± 1.33%) induced by *S*. Typhimurium. *B. coagulans* significantly reduced the binding of *S*. Typhimurium to HT-29 cells (2.69±0.88%) (Fig. 6). Also, the percent of adhesion for *B. coagulans* was measured as 2.69±0.88%.

## DISCUSSION

Salmonellosis has caused a prominent concern in the global economy and as an important public health issue (22). Following bacterial infection, antibiotic resistance is arisen and disturbing the intestinal flora balance (12, 23) which persuaded scientists to focus on other approaches such as probiotics and their metabolites. *In vivo* and *in vitro* studies have shown the promising effects of probiotics in the prevention and/or treatment of salmonella infections (24). The *in vitro* interaction between host cells and foodborne pathogenic bacteria has been previously addressed (25).

In the present study, MIC, effects of *B. coagulans* and its supernatant on the cell cytotoxicity, induction of apoptosis and adhesion caused by *S*. Typhimurium on HT-29 cells were investigated.

The beneficial effects of probiotics exhibit competitive inhibitions with the growth of pathogens such as *Escherichia coli* (26), *Campylobacter jejuni* (27), *Salmonella enterica* serovar Enteritidis (28) and *Clostridium perfringens* (29). According to previous studies, various species of probiotic bacilli can produce bacteriocins. For example, the *Bacillus licheniformis* bacteriocin called Bacillusin 490 that produce at 4°C and at a wide range of pH has antimicrobial activities against pathogens such as *Bacillus smithii, Bacillus anthracis, Bacillus cereus* and *Listeria innocua* (30). Previous studies have also shown that extracts obtained from probiotic *B. subtilis* and *B. coagulans* cultures showed an inhibitory effect on the *Clostridium perfringens* (17, 31). In the present study, the MIC of supernatant obtained from the culture of *B. coagulans* with concentration of 8% showed the highest inhibitory effect on the growth of *S*. Typhimurium. The results showed that by reducing the concentration of *B. coagulans* supernatant in each group, the inhibitory effect was also significantly decreased. This is probably due to the decrease in the concentration of the antimicrobial compounds present in the supernatant.

Higher cytotoxicity was induced by 8% supernatant of *B. coagulans* using the MTT assay. This can be due to the high concentration of toxic compounds produced by *B. coagulans* bacteria. *S*. Typhimurium showed a high level of cytotoxicity. *B. coagulans* was significantly reduced the cytotoxicity induced by *S*. Typhimurium. Different concentrations of *B. coagulans* supernatant did not show any significant difference in reducing the cytotoxicity induced by *S*. Typhimurium. In our previous study, the cytotoxicity of *B. coagulans* and its culture extract on NYSM medium was measured on HT-29 cells, which concluded to similar results to this study (16). The co-culture of *S*. Typhimurium and *B. coagulans* was significantly reduced the cytotoxicity compared to the supernatant. This reduction in cytotoxicity was probably associated with the production of antimicrobial and acid compounds by *B. coagulans*, which inhibit the growth of *S*. Typhimurium and prevent its adhesion to the HT-29 cells.

The results showed that the supernatant of *B. coagulans* with 8 and 6% concentrations in comparison with *B. coagulans* and its supernatant with concentrations of 2 and 4% induced apoptosis in a higher percentage on HT-29 cells. This could be due to an increase in the concentration of toxic compounds and apoptosis induced in the supernatant of this bacterium culture at the concentrations of 6 and 8%.

*B. coagulans* supernatant at concentrations of 4 and 6% did not reduce the induction of apoptosis by *S*. Typhimurium. Simultaneous culture of *S*. Typhimurium with *B. coagulans* supernatant at 8% concentration showed a significantly higher apoptosis rate than *S*. Typhimurium.

*B. coagulans* reduced the percentage of apoptosis induced by *S*. Typhimurium more than other experimental groups. This is probably to prevent adhesion of *S*. Typhimurium to HT-29 cells. The compounds in the supernatant can inhibit the growth of *S*. Typhimurium, but cannot reduce the percentage of apoptosis induced by *S*. Typhimurium.

In this study, the adhesion of *B. coagulans* and *S*. Typhimurium was evaluated. In addition, the effect of *B. coagulans* and its supernatant on the adhesion of *S*. Typhimurium to HT-29 cells was investigated. The results showed that the supernatant with different concentrations did not significantly affected the adhesion of *S*. Typhimurium to HT-29 cells. But the bacterial *B. coagulans*, which also have the ability to adhesion to HT-29 cells, significantly reduced the binding of *S*. Typhimurium to this cell line. In previous studies, the binding rate of *S*. Typhimurium to epithelial cells was measured which was similar to those reported here (15).

In conclusion, *B. coagulans* probiotic and its supernatant can efficiently reduce *S*. Typhimurium injuries to the HT-29 cell line by producing antimicrobial substances. However further surveys should be conducted *in vivo* conditions.
